# Nutritional Status Is Associated with Mortality but Not Appropriate Discharge of Implantable Cardioverter Defibrillators in Patients with Heart Failure

**DOI:** 10.3390/diagnostics15050610

**Published:** 2025-03-04

**Authors:** Idris Yakut, Yücel Kanal, Atik Aksoy, Ozcan Ozeke, Ozgür Ulaş Ozcan, Yasin Ozen, Dursun Aras

**Affiliations:** 1 Department of Cardiology, Medipol İstanbul University, Istanbul 34815, Turkey; ozgurulasozcan@yahoo.com.tr (O.U.O.); drdaras@gmail.com (D.A.); 2 Department of Cardiology, Sivas Cumhuriyet University, Sivas 58140, Turkey; yucel_kanal@hotmail.com; 3 Department of Cardiology, Health Sciences University, Ankara City Hospital, Ankara 06800, Turkey; aksoyatik@gmail.com (A.A.); ozcanozeke@gmail.com (O.O.); 4 Department of Cardiology, Selçuk University, Konya 42130, Turkey; ysnozn70@gmail.com

**Keywords:** CONUT score, heart failure, ICD, malnutrition, PNI

## Abstract

**Objective:** To investigate the predictive value of nutritional status in heart failure (HF) patients with an implantable cardioverter defibrillator (ICD), and to identify factors associated with ICD discharge and mortality. **Methods:** This retrospective study was conducted by analyzing data from 2017 to 2021. HF patients who underwent ICD implantation for primary prevention were included. Follow-up visits were continued until December 2022. Patients were examined based on ICD shock occurrence (ICD-A: appropriate shock), ICD non-discharge (ICD-X), and mortality. Nutritional status was assessed by the Prognostic Nutritional Index (PNI) and the Controlling Nutritional Status (CONUT) scores. **Results:** A total of 221 patients were included in the study, 86 of whom were in the ICD-A group (135 in the ICD-X group). Age and sex distribution were similar in these groups. The all-cause mortality rate was 20.36%. A PNI with a cut-off value of <47.25 and a CONUT score with a cut-off value of >2.5 were able to significantly predict all-cause mortality. The PNI had a greater area under the curve compared to the CONUT. Non-ischemic cardiomyopathy and high left-ventricle end-systolic diameter (ESD) were independently associated with appropriate ICD shock. Low systolic blood pressure, high ESD, low sodium, low total cholesterol, low (<47.25) PNI, and ICD shock were independently associated with all-cause mortality. **Conclusions:** Malnutrition appears to be associated with mortality in patients with primary-prevention ICDs, and the PNI appears to be a more useful indicator than the CONUT for determining the risk of mortality in these patients.

## 1. Introduction

Heart failure, one of the main causes of mortality and morbidity [1,2], is a complex clinical syndrome resulting in systolic and/or diastolic dysfunction [3]. Malnutrition has been demonstrated to increase the frequency of mortality, cardiovascular events, and recurrent hospitalization among patients with heart failure [2,4]. This is a critical relationship since the prevalence of malnutrition in patients with heart failure varies from 16% to 90% [4], potentially due to the fact that factors impacting metabolism (cholesterol, glucose metabolism, etc.) and heart failure itself may lead to loss of appetite, poor digestion, chronic inflammation, reduced absorption, oxidative stress-induced alterations in metabolism, and intensified catabolism [2,3,5]. Furthermore, fluid retention and inflammation are exacerbated by malnutrition, leading to the worsening of heart failure, creating a vicious cycle [4]. Given the high prevalence of malnutrition in individuals with heart failure and its significant impact on prognosis, it becomes imperative to assess malnutrition markers for their prognostic capabilities.

Screening instruments for malnutrition include the Prognostic Nutritional Index (PNI), the Controlling Nutritional Status (CONUT) score, the Global Leadership Initiative on Malnutrition, the Geriatric Nutritional Risk Index and the Mini Nutritional Assessment Score, which are used as prognostic markers in both clinical practice and research [4,6,7,8,9]. The PNI is an indicator of malnutrition that can be easily calculated based on serum albumin and peripheral lymphocyte count, and has been used to evaluate nutritional status in various diseases including heart failure [9,10,11,12]. The Controlling Nutritional Status (CONUT) score, calculated using albumin, peripheral lymphocyte counts, and total cholesterol, is reported to be one of the most reliable markers of nutritional status, especially for hospitalized patients [2,13]. Although the severity of malnutrition assessed by the CONUT or PNI have been associated with the severity of heart failure, the frequency of rehospitalization, and mortality in many studies [2,8,14,15], there are also studies that have not determined relationships between heart failure prognosis and CONUT score or PNI [12,16,17,18].

In heart failure treatment, primary prevention can be achieved by implantable cardioverter defibrillator (ICD) devices, which improve survival by reducing sudden cardiac death in heart failure patients with impaired left ventricular ejection fraction (LVEF) [19]. However, a significant number of patients may not benefit from ICD implantation, making this preventive treatment unnecessary for a large group of individuals. Identifying predictors for patients who will benefit most from an ICD is therefore crucial for patient safety and risk stratification [12].

Malnutrition is a common but often underrecognized condition in heart failure and has been associated with adverse outcomes, including increased mortality and hospitalizations, potentially increasing the risk of arrhythmias through mechanisms such as inflammation, electrolyte imbalances, and myocardial instability. It can contribute to myocardial remodeling, inflammation, and autonomic dysfunction, all of which may increase arrhythmic risk. Despite this, the relationship between nutritional status and ICD dis-charge remains largely unexplored. Most previous studies have focused on general heart failure populations, without distinguishing between patients with and without ICDs [20,21,22,23]. Given that ICDs are primarily used for the prevention of sudden cardiac death due to ventricular arrhythmias, it is unclear whether the mechanisms linking malnutrition to adverse outcomes extend to this specific subgroup. Since ICD therapy does not directly address the systemic and metabolic complications associated with malnutrition, patients with poor nutritional status may still experience high mortality despite receiving this de-vice. While previous studies have linked various clinical factors (sex, LVEF, New York Heart Association [NYHA] class, echocardiographic findings, medications, etc.) to ICD discharge [20,21,22,23], the direct impact of nutritional status on arrhythmic events is not well established. Investigating the relationship between nutritional status and appropriate ICD discharge could provide insights into whether malnourished patients are at higher risk of life-threatening arrhythmic events requiring ICD intervention. Given the potential role of malnutrition in modulating arrhythmic risk, further investigation is warranted to deter-mine whether it influences the effectiveness of ICD therapy.

Therefore, as a primary aim of this study, we aimed to investigate and compare the predictive value of nutritional status (determined by the PNI and the CONUT score) in determining appropriate ICD discharge and mortality in patients with primary-prevention ICD. As a secondary aim, we also sought to identify other factors associated with mortality and appropriate ICD discharge.

## 2. Material and Methods

### 2.1. Ethical Statement, Study Design, and Setting

The study protocol received approval from the Non-invasive Clinical Research Ethics Committee of Istanbul Medipol University (Decision date: 4 January 2024, decision no: 21) and the research was conducted in compliance with the ethical principles outlined in the Declaration of Helsinki and its subsequent amendments. The index application of each patient was defined as the admission due to an arrhythmic event requiring discharge or the first control examination during the study period—given that at least 6 months had surpassed following ICD implantation for primary prevention.

### 2.2. Study Population, Definitions, and Groups

The inclusion criteria were as follows: patients diagnosed with heart failure, those with an LVEF of 40% or less from January 2017 to September 2021, individuals who underwent ICD implantation specifically for primary prevention, and those with at least 6 months elapsed since ICD implantation for primary prevention. Exclusions were applied to patients who did not undergo primary-prevention ICD implantation or underwent it for secondary prevention. Additionally, those with a follow-up period of less than 6 months after primary-prevention ICD implantation, a follow-up period of less than 6 months after the index application, individuals who had a primary-prevention ICD but experienced false discharges, patients with a true discharge before and/or after the index application, and those with missing data were not analyzed. Furthermore, the study excluded individuals with active infections during blood collection, cases with a history of organ transplantation, those undergoing immunosuppressive therapy, individuals who experienced acute coronary syndrome or cardiac surgery within 6 months before the index application, and patients with severe comorbidities such as liver cirrhosis, end-stage renal disease, chronic obstructive pulmonary disease, and cancer.

Shock delivery from an ICD device due to a cardiac event such as ventricular tachycardia or ventricular fibrillation was defined as appropriate ICD discharge. The patients were divided into two groups based on receipt of ICD shock: the ICD-A (appropriate discharge) and the ICD-X (non-discharge) groups. Patients were also divided into two separate groups based on survival: those who died during the follow-up (mortality group) and those who survived (survival group). Sustained ventricular tachycardia and ventricular fibrillation were included in the events requiring discharge.

### 2.3. Data Collection

Age, sex, height, weight, comorbidities, smoking status, laboratory results, diagnosis, and treatment information related to heart failure at the time of hospitalization, and survival status during hospitalization and follow-up were obtained from the computerized database of our hospital and patient charts. Body mass index (BMI) was calculated as weight/height^2^ (kg/m^2^).

### 2.4. Heart Failure Management and Related Instruments

The diagnosis and management of heart failure and ICD implantation were performed according to the most recent guidelines of the European Society of Cardiology and the American College of Cardiology Foundation/American Heart Association. All ICD implantations and follow-up studies were performed at our center. In the case of an ICD discharge (shock), phone follow-up was performed and patients were seen in the clinic for device interrogation and control. All patients were followed up for at least 6 months. Echocardiographic measurements were routinely performed during hospitalization by experienced cardiologists. The following measurements were made according to the American Society of Echocardiography guidelines [24]: EDD, ESD, LVEF, sPAP, TAPSE, and the presence of left-ventricle thrombus. LVEF was measured by the modified Simpson method [25].

Baseline information on the presence of diabetes mellitus, hypertension, atrial fibrillation, cardiomyopathy, systolic blood pressure, LVEF, systolic pulmonary artery pressure (sPAP), left ventricular end-diastolic and end-systolic diameter (EDD and ESD), tricuspid annular plane systolic excursion (TAPSE) values, NYHA classes, ICD implantation status, the presence of left-ventricle thrombus, and cardiac events requiring discharge were screened. Atrial fibrillation was identified by an electrocardiogram performed during index hospitalization. NYHA classification was made as previously described [26].

The determination of cardiomyopathy presence and type relied on specific criteria. Ischemic cardiomyopathy was identified by a reduced LVEF, stemming from (i) occlusion of more than 70% in one or more primary epicardial coronary vessels, (ii) a history of transmural myocardial infarction, (iii) prior coronary procedures (angioplasty, stenting, and/or bypass surgery), or (iv) stress-induced perfusion abnormalities for myocardial ischemia, typically detected through nuclear scintigraphy. In contrast, the presence of non-ischemic cardiomyopathy was based on the detection of low LVEF without valvular abnormalities or discernible ischemic, hypertrophic, or other overt cardiomyopathy etiology [20].

### 2.5. Laboratory Analysis

All laboratory measurements were conducted within the certified laboratories of our hospital, utilizing calibrated standard measuring devices in accordance with the manufacturer’s recommendations. The study encompassed a comprehensive analysis of various parameters obtained from fasting venous blood samples collected during the early morning after overnight fasting at the time of index hospitalization, prior to any prevention. The measured parameters included a complete blood count, encompassing lymphocyte, monocyte, neutrophil, and platelet counts, as well as hemoglobin and hematocrit values. Additionally, the analysis covered glucose, sodium, creatinine, urea, uric acid, aspartate aminotransferase, alanine aminotransferase, albumin, direct bilirubin, total bilirubin, low-density lipoprotein cholesterol (LDL-C), high-density lipoprotein cholesterol, triglyceride, total cholesterol, and brain natriuretic peptide (BNP) levels.

### 2.6. Nutritional Status Assessment Tools

The nutritional status of the patients was determined by PNI and CONUT indices. The PNI is calculated using the following equation: [(10 × serum albumin level (g/dL)) + (0.005 × total peripheral lymphocyte count (per mm^3^))]. A lower PNI score indicates worse nutritional status [8,9]. The CONUT score is calculated by adding serum albumin levels and corresponding score, total peripheral lymphocytes count and score, and total cholesterol levels and score, as described previously [27]. The range of the CONUT score is 0–12, with higher values indicating worse nutritional status [27]. A PNI ≤ 40 [28] and CONUT score ≥ 5 [27] were defined as poor nutritional status.

### 2.7. Follow-Up and Endpoints

The data of the patients were followed up until December 2022 and the last patient included in the study was enrolled 6 months prior to this date. Endpoints were (i) all-cause mortality and (ii) appropriate ICD discharge. All-cause mortality was defined as death from any cause (apart from COVID-19-associated deaths) during the follow-up period regardless of timing (in-hospital or after discharge).

### 2.8. Statistical Analysis

A significance level of *p* < 0.05 was considered for determining statistically significant results. Analyses were conducted using IBM SPSS Statistics for Windows, Version 25.0 (IBM Corp., Armonk, NY, USA). To assess the distribution of numerical variables, histograms and Q–Q plots were employed. Descriptive statistics were presented with mean ± standard deviation for normally distributed continuous variables, with median (25th percentile–75th percentile) for non-normally distributed continuous variables, and with frequency (percentage) for categorical variables. For normally distributed continuous variables, the independent-samples *t*-test was utilized, while the Mann–Whitney U test was employed for non-normally distributed continuous variables. Categorical variables were analyzed using the chi-square test and Fisher’s exact test. The prediction performance of the PNI and CONUT score was assessed through receiver operating characteristic (ROC) curve analysis, with optimal cut-off points determined using the Youden index. Multivariable logistic regression analysis, employing the forward conditional selection method, was conducted to identify factors independently associated with appropriate ICD discharge and all-cause mortality. Variables deemed statistically significant in univariate analysis results were included in the multivariable logistic regression analysis.

## 3. Results

**Figure 1:**
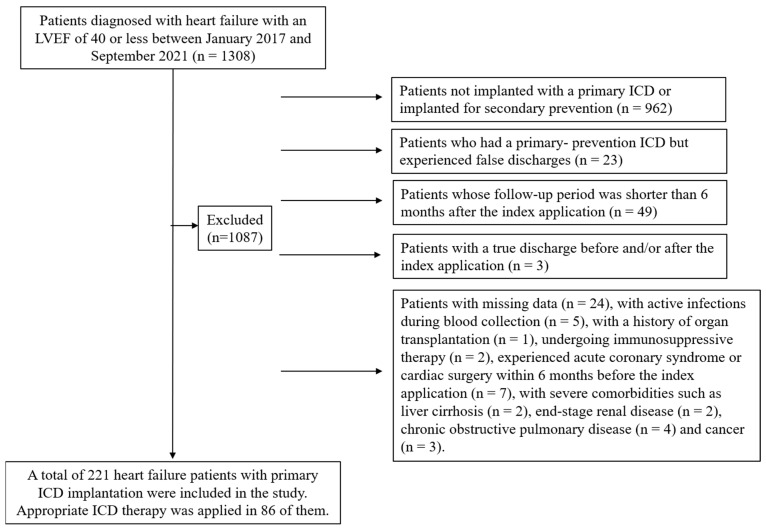
Flow diagram of the study (ICD: implantable cardioverter defibrillator, LVEF: left ventricular ejection fraction).

The data of 1308 patients with potential for inclusion were reviewed. Among these, 293 had undergone ICD implantation for primary prevention. False discharges had been reported in 23 patients. There were 49 patients whose follow-up period was shorter than 6 months. Another 53 patients were excluded based on the remaining exclusion criteria. A final total of 221 patients were included in the study, 86 of whom were in the ICD-A group and 135 in the ICD-X group (Figure 1). All-cause mortality was 20.36%.

Of the 86 patients receiving appropriate ICD discharge, 22 (25.58%) were shocked by anti-tachycardia pacing (ATP), and 64 (74.42%) were shocked for defibrillation purposes. Non-ischemic cardiomyopathy was diagnosed in 102 (46.15%) patients, while 119 (53.85%) patients had ischemic cardiomyopathy. The overall mean age was 52.38 ± 11.47 years, and the ICD-A and ICD-X groups were similar (*p* = 0.200). In terms of sex, 86.43% of all patients (n = 191) were male, and sex distribution was again similar between the ICD-A and ICD-X groups (*p* = 0.381). The ICD-A group had a significantly higher ESD (*p* = 0.001) value and higher frequencies of non-ischemic cardiomyopathy (*p* = 0.001), ventricular tachycardia (*p* < 0.001), and mortality (*p* = 0.040) (Table 1).

According to the PNI, 13 patients (5.88%) were identified as having poor nutritional status, while according to the CONUT, 15 patients (6.88%) were identified as such.

According to multivariable logistic regression analysis, the occurrence of appropriate ICD discharge was independently associated with non-ischemic cardiomyopathy (OR: 2.043, 95% CI: 1.069–3.904, *p* = 0.031) and high ESD (OR: 1.059, 95% CI: 1.021–1.099, *p* = 0.002) (Table 2) but not with nutritional status.

The summary of comparisons between patients with and without mortality are presented in Table 3. Age (*p* = 0.592) and sex distribution (*p* = 0.767) were similar. In the mortality group, the percentage of patients with NHYA class III and IV (*p* < 0.001), atrial fibrillation (*p* = 0.028), appropriate ICD discharge (*p* = 0.040), mean sPAP (*p* = 0.021), and the values for ESD (*p* < 0.001), direct bilirubin, total bilirubin, BNP (*p* < 0.001 for all three), and CONUT score (*p* = 0.001) were significantly higher than the survival group. In contrast, systolic blood pressure (*p* < 0.001), LVEF (*p* = 0.020), TAPSE (*p* < 0.001), lymphocyte count (*p* < 0.001), hemoglobin (*p* = 0.010), hematocrit (*p* = 0.040), sodium (*p* = 0.001), LDL-C (*p* = 0.012), total cholesterol (*p* = 0.007), PNI score (*p* < 0.001), and triglycerides (*p* < 0.001) were significantly lower in those with mortality.

**Figure 2:**
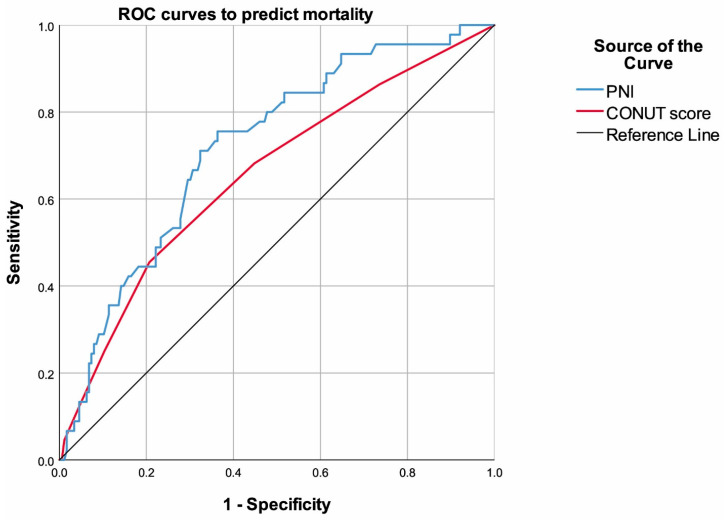
ROC curves of the PNI and CONUT score to predict mortality (CONUT: the Controlling Nutritional Status, PNI: the Prognostic Nutritional Index, and ROC: receiver operating characteristic).

ROC analyses revealed that both PNI and CONUT could predict mortality. Cut-off values were <47.25 (AUC: 0.720, 95% CI: 0.640–0.800) and >2.5 (AUC: 0.656, 95% CI: 0.563–0.749) (Table 4, Figure 2).

According to multivariable logistic regression, mortality was independently associated with low systolic blood pressure (OR: 0.956, 95% CI: 0.922–0.992, *p* = 0.016), high ESD (OR: 1.136, 95% CI: 1.059–1.219, *p* < 0.001), low sodium (OR: 0.782, 95% CI: 0.693–0.884, *p* < 0.001), low total cholesterol (OR: 0.978, 95% CI: 0.963–0.993, *p* = 0.005), low (<47.25) PNI (OR: 6.363, 95% CI: 2.019–20.058, *p* = 0.002), and ICD discharge occurrence (OR: 4.080, 95% CI: 1.302–12.786, *p* = 0.016). Other variables included in the analysis—NYHA classification (*p* = 0.162), atrial fibrillation (*p* = 0.587), LVEF (*p* = 0.326), sPAP (*p* = 0.967), TAPSE (*p* = 0.193), lymphocyte (*p* = 0.844), hemoglobin (*p* = 0.715), hematocrit (*p* = 0.405), direct bilirubin (*p* = 0.232), total bilirubin (*p* = 0.306), LDL-C (*p* = 0.579), triglyceride (*p* = 0.458), BNP (*p* = 0.312), and CONUT score (*p* = 0.511)—were found to be non-significant (Table 5).

## 4. Discussion

The current guidelines recommend the use of ICDs for both primary and secondary prevention with the aim of reducing arrhythmic mortality in patients diagnosed with heart failure and reduced ejection fraction [29]. Despite efforts to identify risk factors for appropriate shock occurrence post-ICD implantation, predicting high-risk patients is challenging, and the role of malnutrition in this context remains unknown [21,22,23,30,31]. In this study, we investigated the relationship between nutritional status (assessed via the PNI and CONUT) and appropriate ICD discharge, in addition to some clinical and laboratory parameters, but neither score was found to be associated with arrhythmic events in patients with primary-prevention ICD. Instead, non-ischemic cardiomyopathy and high ESD were identified as independent variables associated with ICD discharge. These relationships have been investigated in a very limited number of studies. Available evidence from a similar study to ours suggests that PNI is unassociated with appropriate shock in the follow-up of heart failure patients with low LVEF who had ICDs [12]. In the study by Kawasaki et al., no significant relationship was shown between CONUT score and appropriate ICD discharge for severe ventricular tachyarrhythmias [17]. Studies investigating n-3 fatty acids in this context among patients with an ICD have not shown consistent results [32,33]. In a retrospective observational study, the relationship between CONUT score and the response to cardiac resynchronization therapy in patients with advanced-stage heart failure was investigated. Interestingly, the response to resynchronization was significantly lower in patients with moderate or severe malnutrition [2]. There is a large body of publications which have reported factors associated with appropriate ICD discharge. In a study similar to ours, predictors of ICD discharge were listed as nonsustained ventricular tachycardia prior to implant, β-blocker non-use, large EDD, and male sex [20]. The majority of the literature shows that unexplained syncope, maximal left ventricular-wall thickness, nonsustained ventricular tachycardia, abnormal blood pressure response to exercise, family history of sudden cardiac death [34,35], lower LVEF, higher NYHA class [21,22], inducibility of ventricular tachycardia at the time of electrophysiology studies [23], and male sex [30] are reproducible examples of factors associated with the occurrence of ICD activity. The current study found that non-ischemic cardiomyopathy and high ESD may be the most important determinants of ICD prevention, and owing to the large and long-term study design, we believe that these variables should be emphasized in addition to the aforementioned factors in the selection of appropriate ICD candidates. However, the importance of nutritional status in ICD behavior remains uncertain and this issue necessitates more comprehensive and longitudinal studies with longer-term follow-up.

In recent years, there has been a renewed emphasis on identifying patients with heart failure who are at a heightened risk of mortality. There has been a surge in studies which have investigated the correlation between nutritional status and mortality risk in heart failure, but research specifically focused on patients with primary-prevention ICD is notably limited. The results indicate that the PNI with a cut-off value of <47.25 and the CONUT score with a cut-off value of >2.5 demonstrated significant predictive capabilities for mortality in patients with primary-prevention ICD. However, due to the limited accuracy but high negative predictive value, it may be relevant to utilize these scores in the form of screening tests. The high negative predictive value can be utilized to identify patients in which mortality is relatively more unlikely to occur, whereas further testing will be necessary to assess mortality risk in subjects with poor nutritional status. Of note, the PNI exhibited higher sensitivity and AUC compared to the CONUT score, while the specificity of the CONUT score surpassed that of the PNI. Additionally, low PNI also demonstrates an independent relationship with mortality, along with low systolic blood pressure, high ESD, low sodium, low total cholesterol, and the occurrence of ICD discharge. Notably, a study by Çinier et al. also highlighted the association of PNI with long-term all-cause mortality in patients with heart failure and reduced LVEF who underwent ICD implantation [12]. Kawasaki et al. showed that patients with an ICD with a higher CONUT score (≥4) had a higher risk of cardiac death [17]. In a meta-analysis, malnutrition assessed by a CONUT score ≥ 2 was associated with higher risk of all-cause mortality in patients with heart failure. The risk of death from all causes was found to be 1.92 times higher in malnourished heart failure patients. Furthermore, an increase of one point in the CONUT score translated to a 16% higher risk of all-cause mortality [1]. It is also notable that extreme values for malnutrition have a greater impact on outcomes. For instance, Ikeya et al. showed that the mortality rate was significantly higher in patients with moderate or severe malnutrition according to the CONUT score, while a CONUT score of ≥5 was associated with long-term all-cause mortality [2].

Despite the demonstration of relationships between malnutrition and mortality risk, there are also publications that have not found any notable impacts. In a study examining in-hospital and 1-year mortality, the CONUT score did not provide useful prognostic information in detecting the risk of death [16]. Furthermore, the limited diagnostic performance of these scores is exemplified by a prospective study, in which the PNI (cut-off: 39.7) had a sensitivity of 66.1% and specificity of 68.4% in predicting in-hospital mortality, while the CONUT score (cut-off: 5) had a sensitivity of 61.4% and specificity of 68.4%. Nevertheless, the authors reported that both low PNI and high CONUT scores were independent predictors of mortality at 1 year [8]. In another study similar to ours, both the PNI and CONUT were independent predictors of all-cause mortality in patients with heart failure. The ROC analysis showed that the AUC was greater for the PNI compared to the CONUT score [14]. Conflictingly, a prospective study found that the CONUT score was relatively superior in predicting outcomes compared with the PNI [15]. In another study utilizing multivariable analysis, a PNI < 41.2 and a CONUT score > 5 were shown to be independent predictors of in-hospital mortality. In this study, the hazard ratio of the CONUT score was found to be higher than that of the PNI [18]. Apart from nutritional scores, various studies have identified the need for ICD shock, high NYHA class, atrial fibrillation, chronic kidney disease, and diabetes [19,20] as risk factors for mortality in patients with primary-prevention ICD. The possible reasons for the contradictory results concerning the predictive values of the CONUT and PNI include various hypotheses: the inclusion of patients with low or high LVEF, investigation of all-cause mortality or cardiac-related mortality, investigation of in-hospital mortality or long-term mortality, differences in follow-up times, and the presence of acute or chronic heart failure, as well as design-related challenges that include sample size and data collection.

Our findings suggest that while malnutrition remains a strong predictor of mortality in heart failure patients with ICDs, its influence may be mediated through mechanisms beyond arrhythmic risk. Previous studies have demonstrated the prognostic significance of PNI in heart failure, but these studies did not specifically assess patients with ICDs, who have distinct clinical characteristics and risk profiles. The fact that malnutrition was not associated with increased ICD discharges suggests that its impact on prognosis is likely driven by non-arrhythmic mechanisms, such as systemic inflammation, sarcopenia, immune dysfunction, and worsening heart failure. This highlights the need for a broader approach to risk stratification in these patients, which may include incorporation of nutritional assessment as a component of clinical decision-making. Although PNI has been identified as an independent predictor of mortality, its moderate sensitivity and specificity raise concerns about its standalone utility as a risk-stratification tool in clinical practice. However, given that PNI is an easily accessible and calculable parameter, it may contribute to patient management, especially when used in combination with other prognostic indicators. Therefore, further prospective studies are needed to better define the role of the PNI in clinical decision-making. It is notable that the PNI outperforms the CONUT in predicting mortality, especially based on the multivariable results; however, the AUC difference is relatively modest. It may be advisable to assess the PNI further to ascertain its possible role in routine risk assessment, likely in conjunction with other prognostic markers. While the PNI demonstrated a stronger predictive value for mortality, both scores can aid clinicians in risk stratification. Rather than serving as standalone decision-making tools, these indices should be incorporated into a comprehensive patient assessment, particularly when evaluating candidates for ICD implantation. We recommend that systolic blood pressure, ESD, sodium, total cholesterol, and appropriate ICD discharge, along with PNI, be taken into consideration in the risk classification of heart failure patients with ICDs. However, more comprehensive longitudinal studies should be conducted to determine the relationship between nutritional scores and mortality, and also, to determine population-based or universal cut-off values for these scores.

That the association was observed between nutritional indices and mortality, but not with ICD discharges, raises important questions regarding the underlying mechanisms linking nutritional status to outcomes in this population. While arrhythmic events are major contributors to mortality in heart failure patients with ICDs, malnutrition may primarily influence prognosis through relationships that are unassociated with arrhythmia itself. Poor nutritional status is associated with systemic inflammation, muscle wasting, immune dysfunction, and worsening heart failure, all of which contribute to increased mortality risk. These factors may not necessarily trigger ventricular arrhythmias requiring ICD therapy but can lead to progressive cardiac decompensation, infections, and multi-organ dysfunction, ultimately resulting in mortality. This could suggest that nutritional status serves as a marker of overall vulnerability rather than directly influencing arrhythmic burden. Furthermore, heart failure patients with poor nutritional status often exhibit specific hemodynamic phenotypes that are associated with adverse outcomes. The principal hemodynamic phenotypes of heart failure commonly encountered in clinical practice include (1) warm and dry (no signs or symptoms of hypoperfusion or congestion); (2) warm and wet (no hypoperfusion, but presence of congestion); (3) cold and wet (presence of hypoperfusion and congestion); and (4) cold and dry (hypoperfusion without congestion) [36]. Among these, the cold and dry phenotype, which is frequently observed in elderly heart failure patients with poor nutritional status, has been identified as a significant prognostic indicator associated with adverse outcomes [37,38]. Future studies should investigate whether improving nutritional status can reduce non-arrhythmic mortality and whether specific metabolic or inflammatory pathways link malnutrition to worse outcomes in heart failure patients with ICDs.

The study has several limitations. Our study draws these interpretations from retrospectively obtained data from a single center. Despite the large patient count and adherence to best practices regarding patient inclusion, it may only be possible to generalize these findings to similar populations and to patients with similar characteristics. The retrospective design may have also caused the exclusion of various variables that may not have been available in previous years, which may bias the findings. The relatively small sample size, particularly for subgroup analyses of mortality and ICD discharge, may have led to reduced statistical power and limited generalizability of the findings. Although the examined patients had been followed for at least 6 months, which created a good distribution regarding ICD activity, it may have been valuable to utilize longer-term follow-up; however, such a change could have limited patient counts and we believe we have struck a good balance between follow-up duration and endpoint occurrence. Data concerning statin use or other relevant medications were not assessed, and it is evident that some risk factors such as cholesterol levels and hemogram parameters may have been impacted by statins or anti-inflammatory medications. Because these laboratory parameters are used to calculate the CONUT and PNI scores, this omission might affect the reliability of these scores. We also did not assess the longitudinal changes in the CONUT or PNI scores; however, such follow-up would not have provided information regarding the utility of calculating these scores at admission—which constituted the primary question of the study. Since information on the type and brand of ICD devices and mortality etiologies was not available for all patients, these data were not included in the study. Our endpoint includes both defibrillation and appropriate ATP shocks. This means that rather than specifically analyzing the more often life-threatening events of rapid ventricular tachycardia or ventricular fibrillation, we include arrhythmic events that are likely to consist of slower, more stable ventricular tachycardia.

## 5. Conclusions

In summary, our investigation into heart failure patients with primary-prevention ICDs has yielded insights into the predictive capabilities of nutritional indices. Neither the PNI nor the CONUT were associated with ICD activity; however, both scores demonstrated moderate associations with mortality, especially owing to their high negative predictive values. In particular, the PNI was identified as an independent predictor for mortality among heart failure patients with ICDs, alongside parameters such as systolic blood pressure, ESD, sodium, total cholesterol, and the occurrence of ICD discharges. This comprehensive analysis suggests a link between malnutrition and mortality in primary-prevention ICD patients, evidently with PNI as the more reliable indicator relative to CONUT. The study also reveals that evidence concerning the malnutrition–ICD discharge relationship is limited and contradictory, indicating the need for further research.

## Figures and Tables

**Table 1:** Summary of variables with regard to ICD discharge (shock).

		Appropriate ICD Discharge		
	Total (n = 221)	No (n = 135)	Yes (n = 86)	*p*
Age, years	52.38 ± 11.47	53.17 ± 11.45	51.14 ± 11.45	0.200
Sex				
Female	30 (13.57%)	21 (15.56%)	9 (10.47%)	0.381
Male	191 (86.43%)	114 (84.44%)	77 (89.53%)	
Height, cm	172.55 ± 6.86	172.66 ± 7.11	172.38 ± 6.48	0.763
Weight, kg	81.48 ± 13.38	82.72 ± 13.47	79.51 ± 13.06	0.084
Body mass index, kg/m^2^	27.29 ± 3.65	27.67 ± 3.60	26.69 ± 3.67	0.054
Smoking status				
Non-smoker	92 (44.44%)	57 (46.34%)	35 (41.67%)	0.513
Smoker	67 (32.37%)	36 (29.27%)	31 (36.90%)	
Ex-smoker	48 (23.19%)	30 (24.39%)	18 (21.43%)	
Digoxin use	67 (30.32%)	42 (31.11%)	25 (29.07%)	0.748
NYHA classification				
Class I	18 (8.14%)	13 (9.63%)	5 (5.81%)	0.775
Class II	128 (57.92%)	76 (56.30%)	52 (60.47%)	
Class III	65 (29.41%)	40 (29.63%)	25 (29.07%)	
Class IV	10 (4.52%)	6 (4.44%)	4 (4.65%)	
Diabetes mellitus	75 (33.94%)	46 (34.07%)	29 (33.72%)	0.957
Hypertension	103 (46.61%)	60 (44.44%)	43 (50.00%)	0.420
Atrial fibrillation	84 (38.01%)	48 (35.56%)	36 (41.86%)	0.346
Cardiomyopathy				
Non-ischemic	102 (46.15%)	54 (40.00%)	48 (55.81%)	**0.021**
Ischemic	119 (53.85%)	81 (60.00%)	38 (44.19%)	
Systolic blood pressure (mmHg)	116.30 ± 16.73	117.52 ± 15.84	114.40 ± 17.97	0.177
LVEF, %	23.43 ± 6.29	23.86 ± 6.20	22.76 ± 6.41	0.204
sPAP (mmHg)	41.87 ± 12.05	41.88 ± 11.97	41.87 ± 12.24	0.997
EDD, mm	63.09 ± 10.30	62.04 ± 10.03	64.70 ± 10.55	0.063
ESD, mm	52.96 ± 9.77	50.91 ± 9.57	56.03 ± 9.32	**0.001**
TAPSE, mm	16.14 ± 3.67	16.02 ± 3.77	16.34 ± 3.51	0.581
LV thrombus	19 (8.60%)	10 (7.41%)	9 (10.47%)	0.586
Neutrophil (×10^3^)	5.25 (4.00–6.60)	5.13 (4.10–6.40)	5.41 (3.97–6.82)	0.593
Lymphocyte (×10^3^)	1.96 ± 0.74	1.95 ± 0.68	1.98 ± 0.82	0.775
Monocyte (×10^3^)	0.55 (0.45–0.70)	0.56 (0.46–0.72)	0.53 (0.44–0.67)	0.404
Hemoglobin, g/dL	14.07 ± 1.91	14.11 ± 1.83	14.02 ± 2.04	0.726
Hematocrit, %	43.26 ± 5.42	43.47 ± 5.24	42.94 ± 5.71	0.473
Platelet (×10^3^)	237.28 ± 78.30	242.84 ± 79.16	228.44 ± 76.55	0.185
Glucose, mg/dL	102 (90–130)	105 (89–131)	102 (92–128)	0.761
Sodium, mEq/L	137.69 ± 4.44	137.58 ± 4.48	137.86 ± 4.38	0.646
Creatinine, mg/dL	1.01 (0.88–1.14)	1.00 (0.88–1.14)	1.05 (0.86–1.14)	0.650
Urea, mg/dL	40 (30–52.1)	41 (31–52.19)	37.5 (29–52)	0.251
Uric acid, mg/dL	7.05 ± 2.18	7.22 ± 2.24	6.80 ± 2.08	0.170
AST, IU/L	23 (18–30)	23 (19–31)	22 (18–29)	0.583
ALT, IU/L	24 (16–34)	25.5 (16–35)	20 (15–31)	0.113
Albumin, g/dL	3.79 ± 0.35	3.77 ± 0.34	3.83 ± 0.37	0.260
Direct bilirubin, mg/dL	0.24 (0.17–0.42)	0.23 (0.20–0.40)	0.26 (0.16–0.45)	0.927
Total bilirubin, mg/dL	0.80 (0.59–1.26)	0.80 (0.58–1.22)	0.80 (0.60–1.30)	0.985
LDL-C, mg/dL	97.59 ± 34.71	96.65 ± 35.46	99.03 ± 33.69	0.622
Triglyceride, mg/dL	133 (92–187)	135 (92–179)	119 (88–193)	0.673
HDL-C, mg/dL	39 (33–45)	38 (32–43)	40 (34–48)	0.096
Total cholesterol, mg/dL	168.80 ± 44.46	167.78 ± 46.20	170.36 ± 41.86	0.676
BNP, pg/mL	891 (293–2170)	908 (227–2170)	850.1 (339.8–2162)	0.783
PNI score	47.71 ± 4.94	47.43 ± 4.61	48.14 ± 5.42	0.304
CONUT score	1 (1–3)	1 (1–3)	2 (0–2)	0.958
Mortality	45 (20.36%)	21 (15.56%)	24 (27.91%)	**0.040**

Descriptive statistics were presented by using mean ± standard deviation for normally distributed continuous variables, median (25th percentile–75th percentile) for non-normally distributed continuous variables, and frequency (percentage) for categorical variables. Abbreviations: ALT: alanine aminotransferase, AST: aspartate aminotransferase, BNP: brain natriuretic peptide, CONUT: the Controlling Nutritional Status, EDD: Left ventricular end-diastolic diameter, ESD: Left ventricular end-systolic diameter, HDL-C: High-density lipoprotein cholesterol, ICD: Implantable cardioverter defibrillator, LDL-C: Low-density lipoprotein cholesterol, LV: Left ventricle, LVEF: Left ventricular ejection fraction, NYHA: New York Heart Association, PNI: the Prognostic Nutritional Index, sPAP: systolic pulmonary artery pressure, and TAPSE: tricuspid annular plane systolic excursion. Bold: *p* < 0.05.

**Table 2:** Significant factors independently associated with ICD discharge—multivariable logistic regression analysis.

	β Coefficient	Standard Error	95% CI for β Coefficient		*p*	Exp(β)	95% CI for Exp(β)	
Cardiomyopathy, Non-ischemic	0.714	0.331	0.061	1.367	**0.031**	2.043	1.069	3.904
ESD	0.057	0.019	0.019	0.095	**0.002**	1.059	1.021	1.099
Constant	−3.774	1.032	−5.811	−1.737	<0.001			

Nagelkerke R^2^ = 0.127. Abbreviations: CI: confidence interval, ESD: left ventricular end-systolic diameter, and ICD: implantable cardioverter defibrillator. Bold: *p* < 0.05.

**Table 3:** Summary of variables with regard to mortality.

	Mortality		
	No (n = 176)	Yes (n = 45)	*p*
Age, years	52.17 ± 11.89	53.20 ± 9.72	0.592
Sex			
Female	25 (14.20%)	5 (11.11%)	0.767
Male	151 (85.80%)	40 (88.89%)	
Height, cm	172.86 ± 6.90	171.34 ± 6.63	0.191
Weight, kg	82.29 ± 13.31	78.25 ± 13.31	0.073
Body mass index, kg/m^2^	27.47 ± 3.67	26.56 ± 3.50	0.137
Smoking status			
Non-smoker	71 (43.29%)	21 (48.84%)	0.752
Smoker	55 (33.54%)	12 (27.91%)	
Ex-smoker	38 (23.17%)	10 (23.26%)	
Digoxin use	51 (28.98%)	16 (35.56%)	0.500
NYHA classification			
Class I	17 (9.66%)	1 (2.22%)	**<0.001**
Class II	112 (63.64%)	16 (35.56%)	
Class III	44 (25.00%)	21 (46.67%)	
Class IV	3 (1.70%)	7 (15.56%)	
Diabetes mellitus	59 (33.52%)	16 (35.56%)	0.936
Hypertension	88 (50.00%)	15 (33.33%)	0.067
Atrial fibrillation	60 (34.09%)	24 (53.33%)	**0.028**
Cardiomyopathy			
Non-ischemic	80 (45.45%)	22 (48.89%)	0.807
Ischemic	96 (54.55%)	23 (51.11%)	
Systolic blood pressure	118.77 ± 15.74	106.67 ± 17.16	**<0.001**
LVEF	23.93 ± 6.09	21.49 ± 6.74	**0.020**
sPAP, mmHg	40.91 ± 12.31	45.59 ± 10.29	**0.021**
EDD, mm	62.41 ± 9.39	65.70 ± 12.99	0.057
ESD, mm	51.44 ± 9.37	58.51 ± 9.30	**<0.001**
TAPSE, mm	16.81 ± 3.50	14.12 ± 3.44	**<0.001**
LV thrombus	14 (7.95%)	5 (11.11%)	0.551
Neutrophil (×10^3^)	5.17 (4.04–6.30)	5.54 (3.81–7.60)	0.266
Lymphocyte (×10^3^)	2.07 ± 0.74	1.53 ± 0.57	**<0.001**
Monocyte (×10^3^)	0.53 (0.45–0.70)	0.60 (0.41–0.70)	0.641
Hemoglobin, g/dL	14.24 ± 1.92	13.42 ± 1.74	**0.010**
Hematocrit, %	43.64 ± 5.53	41.78 ± 4.76	**0.040**
Platelet (×10^3^)	238.41 ± 78.78	232.84 ± 77.11	0.671
Glucose, mg/dL	105 (89.5–129.5)	99 (93–135)	0.920
Sodium, mEq/L	138.44 ± 3.40	134.76 ± 6.43	**0.001**
Creatinine, mg/dL	1.01 (0.88–1.14)	1.03 (0.88–1.14)	0.866
Urea, mg/dL	39.5 (29.5–52.05)	44 (34–57)	0.139
Uric acid, mg/dL	6.92 ± 2.10	7.61 ± 2.44	0.061
AST, IU/L	22 (18–30)	24 (20–29)	0.266
ALT, IU/L	24 (16–34)	20 (15–31)	0.413
Albumin, g/dL	3.81 ± 0.36	3.72 ± 0.33	0.142
Direct bilirubin, mg/dL	0.21 (0.16–0.34)	0.37 (0.26–0.72)	**<0.001**
Total bilirubin, mg/dL	0.73 (0.56–1.18)	1.02 (0.72–1.78)	**<0.001**
LDL-C, mg/dL	100.54 ± 35.50	85.88 ± 28.90	**0.012**
Triglyceride, mg/dL	138.5 (97–201)	98 (74.5–138)	**<0.001**
HDL-C, mg/dL	38.5 (34–44)	40 (32–46.5)	0.859
Total cholesterol, mg/dL	172.83 ± 43.61	152.86 ± 44.70	**0.007**
BNP, pg/mL	625 (184–1505)	2162 (831.5–3373)	**<0.001**
PNI	48.43 ± 4.88	44.89 ± 4.15	**<0.001**
CONUT score	1 (0–2)	2 (1–3.5)	**0.001**
Appropriate ICD discharge	62 (35.23%)	24 (53.33%)	**0.040**

Bold is for significance *p* < 0.05. Descriptive statistics were presented by using mean ± standard deviation for normally distributed continuous variables, median (25th percentile–75th percentile) for non-normally distributed continuous variables, and frequency (percentage) for categorical variables. Abbreviations: ALT: alanine aminotransferase, AST: aspartate aminotransferase, BNP: brain natriuretic peptide, CONUT: the Controlling Nutritional Status, EDD: left ventricular end-diastolic diameter, ESD: left ventricular end-systolic diameter, HDL-C: high-density lipoprotein cholesterol, ICD: implantable cardioverter defibrillator, LDL-C: low-density lipoprotein cholesterol, LV: left ventricle, LVEF: left ventricular ejection fraction, NHYA: New York Heart Association, PNI: the Prognostic Nutritional Index, sPAP: systolic pulmonary artery pressure, and TAPSE: tricuspid annular plane systolic excursion.

**Table 4:** Performance of PNI and CONUT score to predict mortality.

	PNI	CONUT Score
Cut-off	<47.25	>2.5
Sensitivity	75.56%	45.45%
Specificity	63.64%	79.31%
Accuracy	66.06%	72.48%
PPV	34.69%	35.71%
NPV	91.06%	85.19%
AUC (95% CI)	0.720 (0.640–0.800)	0.656 (0.563–0.749)
*p*	**<0.001**	**0.001**

Abbreviations: AUC: area under the ROC curve, CI: confidence interval, CONUT: the Controlling Nutritional Status, NPV: negative predictive value, PNI: the Prognostic Nutritional Index, and PPV: positive predictive value. Bold is for significance *p* < 0.05.

**Table 5:** Significant factors independently associated with mortality—multivariable logistic regression analysis.

	β Coefficient	Standard Error	95% CI for β Coefficient		*p*	Exp(β)	95% CI for Exp(β)	
Systolic blood pressure	−0.045	0.018	−0.081	−0.009	**0.016**	0.956	0.922	0.992
ESD	0.127	0.036	0.056	0.198	**<0.001**	1.136	1.059	1.219
Sodium	−0.245	0.062	−0.367	−0.123	**<0.001**	0.782	0.693	0.884
Total cholesterol	−0.022	0.008	−0.038	−0.006	**0.005**	0.978	0.963	0.993
PNI, <47.25	1.851	0.586	0.694	3.008	**0.002**	6.363	2.019	20.058
ICD shock	1.406	0.583	0.255	2.557	**0.016**	4.080	1.302	12.786
Constant	31.742	8.483	14.991	48.493	<0.001			

Nagelkerke R^2^ = 0.609. Abbreviations: CI: confidence interval, ESD: left ventricular end-systolic diameter, ICD: implantable cardioverter defibrillator, and PNI: the Prognostic Nutritional Index. Bold is for significance *p* < 0.05.

## Data Availability

The raw data supporting the conclusions of this article will be made available by the authors upon request.
